# Protective Effectiveness of Hantavirus Vaccine

**DOI:** 10.3201/eid1012.040684

**Published:** 2004-12

**Authors:** Keeho Park, Chang Soo Kim, Ki-Tae Moon

**Affiliations:** * National Cancer Center, Goyang, Republic of Korea;; † Republic of Korea Army, Gyeryong, Republic of Korea;; ‡ Yonsei University College of Medicine, Seoul, Republic of Korea

**Keywords:** hantavirus vaccine, hemorrhagic fever with renal syndrome, effectiveness, dispatch

## Abstract

A case-control study in the Republic of Korea evaluated the protective effectiveness of the hantavirus vaccine. Point estimates showed increasing effectiveness with increasing numbers of doses received: 25% for one dose, 46% for two doses, and 75% for three doses. All 95% confidence intervals overlapped zero; therefore, the findings could be due to chance.

In 1990, the Republic of Korea (ROK) approved a vaccine against the Hantaan virus after accepting data that showed a high seroconversion rate as a surrogate for vaccine effectiveness (*1*). The recommended schedule for vaccination is two doses 1 month apart, as a primary vaccination, and one booster 12 months later. Although the hantavirus vaccine has been in use since approval, and millions of doses have been given, the effectiveness of the vaccine continues to be debated. However, protective effectiveness of the hantavirus vaccine has been measured mainly by serologic studies (*2**–**4*).

The Korean Army is one of the largest consumers of the hantavirus vaccine, second only to public health centers. Uncertainty about protective effectiveness of the vaccine has been enhanced by reports on military personnel in whom hemorrhagic fever with renal syndrome (HFRS) developed, even though they had received the vaccine.

The ROK Army defines a "high-risk area" for HFRS as an administrative district where HFRS cases have occurred during the previous 3 years. Vaccination programs focus on military units located in these high-risk areas, but vaccinating all personnel in those units is impossible because of budget limitations. Therefore, coverage is not 100%.

In a recent study (*4*), the authors noted that since a vaccination campaign began in 1991, the number of HFRS patients has decreased significantly (Figure 1). However, vaccination is likely not the only factor affecting secular trends in number of HFRS patients; climatic and environmental changes also likely play a role. The present case-control study was conducted to assess the protective effectiveness of the hantavirus vaccine.

**Figure 1:**
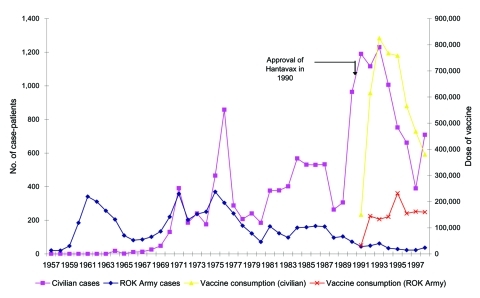
Secular trends in the numbers of hemorrhagic fever with renal syndrome cases, Republic of Korea (ROK), 1957–1998.

## The Study

Cases were identified through the hospital-based active surveillance system of the HFRS maintained by the Korean Army. Cases occurring from January 1, 2002, to January 1, 2004, were enrolled prospectively. For early detection of HFRS, the Korean Army used the operational clinical criteria to identify cases. Patients with HFRS may have sudden onset of fever; experience pain in the head, abdomen, and lower back; and report bloodshot eyes and blurry vision. Petechiae may appear on the upper body and soft palate. The patient's face, chest, abdomen, and back often appear flushed and red, as if sunburned. A confirmed case of HFRS is defined as a positive result on the high-density particle agglutination test.

For each case, one control was selected from among the other patients at each hospital where the case-patient had been hospitalized. The control was matched with the case-patient according to unit, age at the time of hospitalization (±3 years), date of hospitalization (±3 months), and date of transfer to the present unit (±3 months). If no suitable control could be found, the intervals around the case-patient's unit, age, date of hospitalization and date of transference were progressively widened until one or more potential controls were found. For each case-patient, all eligible controls were listed, and one suitably matched control was identified at random. As with the case-patients, the final decisions about each control patient's eligibility for the study were made on the basis of a detailed review of hospital records. Decisions about the eligibility of potential controls were made without knowledge of their vaccination status.

History of vaccination was sought from vaccination records kept at each unit. Vaccine had to be received at least 3 weeks before hospitalization because of the time required for antibodies to develop and because the incubation period is ≈3 weeks on average (*5*). One patient vaccinated <3 weeks before hospitalization was excluded from the data analysis.

Estimates of the relative odds of HFRS associated with vaccination were estimated by using methods developed by Mantel and Haenszel (*6*), which are appropriate for matched designs. The protective effectiveness of the vaccine was estimated as 1 minus the relative odds associated with vaccine use, times 100. Ninety-five percent confidence limits for the effectiveness were derived from the 95% confidence interval (CI) of the relative odds. Data were analyzed with SPSS, version 10.0 (SPSS, Inc., Chicago, IL).

**Figure 2:**
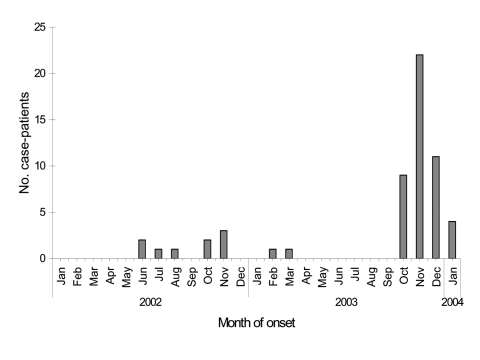
Hemorrhagic fever with renal syndrome cases among Republic of Korea military personnel, by month of onset, January 2002 to January 2004.

From January 1, 2002, to January 1, 2004, a total of 57 HFRS cases were identified among troops of the Korean Army. Of the 57 patients, 3 (5.3%) died. One of three deaths occurred in previously healthy, vaccinated (first and second doses) personnel. Twelve, 9, and 2 cases occurred in personnel who were vaccinated with one, two, and three doses, respectively. Most cases occurred in October (15.7%), November (35.7%), and December (17.1%), although disease also occurred during the spring and the summer (Figure 2).

Of the 54 persons identified with HFRS from January 2002 to January 2004, those who were vaccinated within 3 weeks of hospitalization were excluded from analysis. Because the effectiveness was calculated by comparing each dose (exactly one to three) with no vaccination, case-patients or controls not applicable to that comparison were excluded from each matched set. Finally, 41, 38, and 31 matched sets were formed for one, two, and three doses of the hantavirus vaccine, respectively (Table). Ages of the case-patients were similar to ages of controls. Estimates of vaccine effectiveness according to the number of doses received rose from 25% (95% CI –78% to 68%) for one dose to 46% (95% CI –35% to 78%) for two doses to 75 % (95% CI –18% to 95%) for three doses. When recipients for whom 1 year had passed since their second dose were excluded, effectiveness of two doses increased markedly to 70% (95% CI –9% to 92%).

**Table:** Characteristics of patients and matched controls^a^

Characteristic	No. doses (matched sets)					
	3 (31)		2 (38)		1 (41)	
	Patients	Controls	Patients	Controls	Patients	Controls
Median age (y) (mean ± SD)	22.0 (23.2 ± 3.5)	22.0 (22.0 ± 3.6)	22.0 (23.2 ± 3.1)	21.0 (22.0 ± 2.1)	22.0 (23.4 ± 3.8)	21.0 (22.2 ± 0.7)
% vaccinated	6.5	32.3	23.7	39.5	29.3	36.6

^a^All participants were men.

## Conclusions

The results of this study suggest a trend toward protection for the hantavirus vaccine. The protective effectiveness of the vaccine strongly depends on the number of doses. In particular, effectiveness increased when persons for whom >1 year had passed since their second dose were excluded, which suggests that the protective effect of the second primary vaccination does not persist beyond the period recommended for having the booster dose. In addition, we do not know whether the recommended immunization schedule was optimal for military personnel and farmers, groups for whom hantavirus vaccination is recommended. The vaccination schedule should be epidemiologically relevant, immunologically effective, operationally feasible, and socially acceptable (*7*).

In a field study from the former republic of Yugoslavia conducted by Korean researchers, including the developers of the hantavirus vaccine (*8*), no case of HFRS was observed among 1,900 vaccinees, while 20 confirmed cases were observed among 2,000 nonvaccinated controls. Considering that our study showed low protective effectiveness for one or two doses, that no case of HFRS occurred in Yugoslavian vaccinees before they received the full three doses was surprising.

Because the case-control studies were not experimental, they may be subject to biases. The most important potential biases that might affect this kind of study are detection and selection bias. If all cases of HFRS were identified, no detection bias would occur. Because patients were identified prospectively by active surveillance, we believe that virtually all cases of HFRS diagnosed during the study period were identified. Selection bias may occur when controls do not represent the general population. In this study, controls were selected randomly from a list of potentially eligible controls by using a systemic algorithm. Confounding influences affect the results of a case-control study if controls differ from case-patients in characteristics related to risk of contracting the disease and likelihood of receiving the vaccine. Since the population of this study consisted of military personnel, bias due to sociodemographic differences may be negligible. Therefore, other candidate confounding factors determined by considering the military milieu were used as matching variables.

We could not show that vaccine effectiveness estimates were significant. All of our confidence intervals have lower bounds less than zero. Therefore, while point estimates show effectiveness, this finding could be due to chance. Of course, the range of point estimates in studies with relatively small samples can be wide, and wide confidence intervals that include zero are not uncommon in many studies on vaccine effectiveness (*9*). However, caution is appropriate in interpreting our estimates of vaccine effectiveness.

Finally, this study represents a short-term (7.3 months average) evaluation of protective effectiveness of three doses of the hantavirus vaccine. To assess the long-term effectiveness, protection must be monitored over a longer period.
